# Clinical and non-clinical healthcare workers faced similar risk of acquiring 2009 pandemic H1N1 infection

**DOI:** 10.1186/1753-6561-5-S6-P80

**Published:** 2011-06-29

**Authors:** PTY Ching, CHS Lam, BJ Cowling, WH Seto

**Affiliations:** 1 Hospital Authority, School of Public Health, The University of Hong Kong, Hong Kong, China; 2 Infectious Disease Epidemiology Group, School of Public Health, The University of Hong Kong, Hong Kong, China

## Introduction / objectives

In the 2009 H1N1 pandemic, the Hospital Authority managing >90% of hospital beds and 74 outpatient clinics in Hong Kong implemented mandatory reporting for healthcare workers (HCWs) with confirmed pH1N1 and collected detailed data on infected HCWs.

## Methods

Under mandatory reporting, HCWs with influenza-like illness must present themselves to staff clinic and tested for pH1N1 by RT-PCR and viral culture. A confirmed case was defined as positive on either test. A standard questionnaire was used to assess clinical presentation and nature of exposure. Clinical staff were defined as HCWs involved in direct patient care and non-clinical staff are those without. The reporting for all staff began on 17 June until 31 August 2009. From 1 September 2009, it was mandatory only for clinical staff until 31 May 2010 when the pandemic was downgraded Infection control guidelines were issued on 29 April 2009 and education sessions were attended by >39,000 staff.

## Results

During staff mandatory reporting, there were 249 confirmed pH1N1 cases among 40,511 clinical staff (0.62%) and 119 among 18,759 non-clinical staff (0.63%; p=0.82). The relative risk for clinical versus non-clinical staff was 0.98 (95% CI, 0.78-1.20). In the entire reporting period, a total of 1039 (2.6%) clinical staff had pH1N1 infection. Among clinical staff, 212/1039 (20%) cases reported contact with a confirmed pH1N1 infection, similar to the 24/119 (20%) from non-clinical staff. Importantly, unprotected exposure to a colleague confirmed with pH1N1 were 10-fold higher than exposure to infected patients, similar for clinical (9%) and non-clinical (8.4%) staff (p = 0.97).

## Conclusion

Attack rate was similar for clinical and non-clinical staff showing no increased risk in clinical care.

## Disclosure of interest

P. Ching: None declared, C. Lam: None declared, B. Cowling Grant/Research support from MedImmune Inc., W. H. Seto Other Presented in anti infective meeting 2010 by Pfizer.

